# Factitious Disorder: An Angioedema Copycat

**DOI:** 10.7759/cureus.25638

**Published:** 2022-06-03

**Authors:** Sunydip Gill, Dmitrii Malnev, Jilmil S Raina

**Affiliations:** 1 Medicine, St. George's University School of Medicine, Brooklyn, USA; 2 Internal Medicine, Brookdale University Hospital Medical Center, Brooklyn, USA

**Keywords:** c1 esterase inhibitor, allergy and anaphylaxis, anaphylaxis, angioedema, factitious disorder

## Abstract

Factitious disorder (FD) is a psychiatric illness in which an individual assumes the role of a patient by manifesting physical or psychological symptoms without conscious or obvious reward. Here, we present the case of a 28-year-old female with a history of endotracheal intubations 19 times secondary to anaphylaxis. During the current hospital visit, she complained of cough, shortness of breath (SOB), arthralgia, wheezing, and rashes over the chest. Serum C1 esterase inhibitor and C4 levels have been negative on multiple occasions. A previous laryngoscopy showed a normal larynx, normal vocal cords, and no obstruction. Due to the patient’s history of multiple invasive procedures, malingering was considered a possible differential diagnosis. The patient also has a past psychiatric history of major depressive disorder (MDD), post-traumatic stress disorder (PTSD), adjustment disorder with anxious mood, and anxiety disorder. Her complicated psychiatric history coupled with her multiple endotracheal intubations associated with normal laboratory findings raise the suspicion of factitious disorder. This case is meant to demonstrate the complicated matter of helping a patient whose psychiatric illnesses have put her at risk of serious health complications for the sake of assuming a sick role.

## Introduction

Angioedema is a form of asymmetric swelling of the deep layers of the skin that is mediated by several mechanisms, including histamine and bradykinin [1]. It can be categorized into several subcategories, with allergic, idiopathic, genetic, acquired, or drug-related being used to distinguish unique clinical manifestations. Factitious disorder (FD), as defined by the Diagnostic and Statistical Manual of Mental Disorders, Fifth Edition (DSM-V), is a psychiatric condition in which an individual assumes the role of a patient, either via fabricated physical or psychological issues, without apparent external reward. Being a young, single adult female, with a history of hospitalizations and traumatic relationships, is reported as a risk factor. Factitious angioedema can mimic a life-threatening allergic reaction that requires immediate medical attention [2]. There have been multiple reported cases of factitious angioedema reported in the literature [3]. Here we present the case of a 28-year-old female with numerous hospitalizations over the course of her adult life for episodes of angioedema that required endotracheal intubations without definitive evidence of positive laboratory studies, allergy panels, and a positive psychological history of major depressive disorder, post-traumatic stress disorder (PTSD), adjustment disorder with anxious mood, and anxiety disorder.

## Case presentation

Our patient is a 28-year-old morbidly obese female (BMI 36.6 kg/m^2^) with a past medical history of multiple allergies and multiple endotracheal intubations (19 times). Past medical history was significant for gastric bypass surgery, asthma, deep venous thrombosis (DVT), seizure disorder, major depression disorder, anxiety, and PTSD. Her mother confirmed that our patient had an extensive psychiatry history of MDD, anxiety, and PTSD with multiple psychosocial stressors. The patient reported an allergy to all fruits, ibuprofen, clindamycin, iodides, ketorolac, nitrofurantoin, peanuts, penicillin’s, tree nuts, vegetables, Bactrim, and Alsoy® soy formula (Nestlé S.A., Vevey, Switzerland). She reports an anaphylactic reaction to the above-mentioned agents.

At this admission, she presented to the emergency department (ED) with hoarseness and throat swelling. Our patient was confirmed to have an extensive psychiatry history with MDD, anxiety, and PTSD together with multiple psychosocial stressors according to her mother. She took her epinephrine pen at home without any relief, so she came to ED. On arrival, the patient was dyspneic to 24 breaths per minute with severe stridor, swollen uvula in the posterior pharynx, and was only able to speak two to three words. On examination, the patient was having tachycardia to 102 beats per minute, wheezing and stridor on auscultation, swollen uvula, and narrowing of posterior pharynx noted. The patient received epinephrine 0.3 mg intramuscular (IM) x4, dexamethasone 20 mg intravenous (IV), racemic epinephrine x2, albuterol x2, and diphenhydramine 50 mg x2. Due to persistent respiratory distress despite maximum conservative treatment, the patient was intubated for impending airway closure. The patient was admitted to critical care medicine (CCM) and subsequently transferred to the medical intensive care unit (MICU).

**Figure 1:**
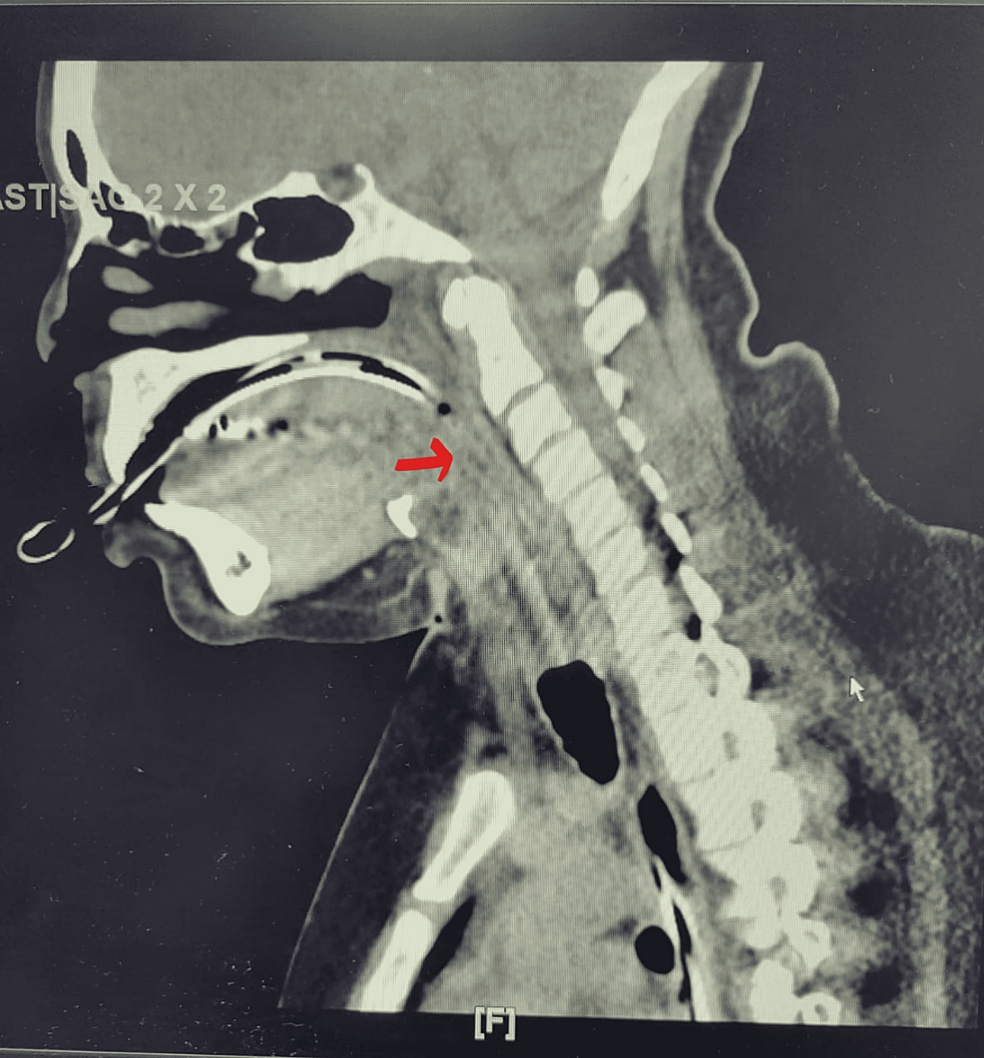
There are indwelling endotracheal and nasogastric tubes. There is a complete collapse of the airway along the tubes. Airway structures cannot be adequately assessed in this setting. The arrow shows the collapse of the airway along the tubes.

The serum C1 esterase inhibitor was 25 mg/dl on admission (reference range 16-33 mg/dl). Her food allergy profile was negative. Serum values of complement C3 and C4, CH50, C1 esterase inhibitor, and histamine level on multiple occasions prior to admission as per the chart review were all within normal levels. Computed tomography (CT) of the soft tissue of the neck showed the complete collapse of the airway along indwelling tubes. Airway structures and retropharyngeal structures could not be adequately assessed (Figure 1).

The patient received Benadryl 25 mg IV every six hours, methylprednisolone 60 mg IV every six hours, and montelukast 10 mg nightly. Due to the concern of tracheobronchomalacia (TBM), bronchoscopy through an endotracheal tube (ET) was performed. On visual inspection, the patient had a patent airway on inspiration and expiration and no sign of narrowing or TBM was noted. The patient was later successfully extubated; no stridor was noted on exam, post extubating.

After extubating, she was transferred to the regular medicine floor. She continued complaining of hoarseness, difficulty breathing, inability to eat due to nausea, and feeling that her throat is closing. At the same time, the patient was able to speak in full sentences, there was no stridor but mild wheezing was noted on examination, and the patient was seen multiple times ordering food outside of the hospital and consuming it without any difficulty. The patient requested to increase diphenhydramine to 75 mg every eight hours and stated it as being her home dose, however, it was above maximum dosing and the patient was receiving 50 mg every 6 hours already. In multiple instances, the CT neck scan was rescheduled, as the patient refused to undergo the test, claiming she was eating but explaining on morning rounds that breathing troubles prevented her from eating.

**Figure 2:**
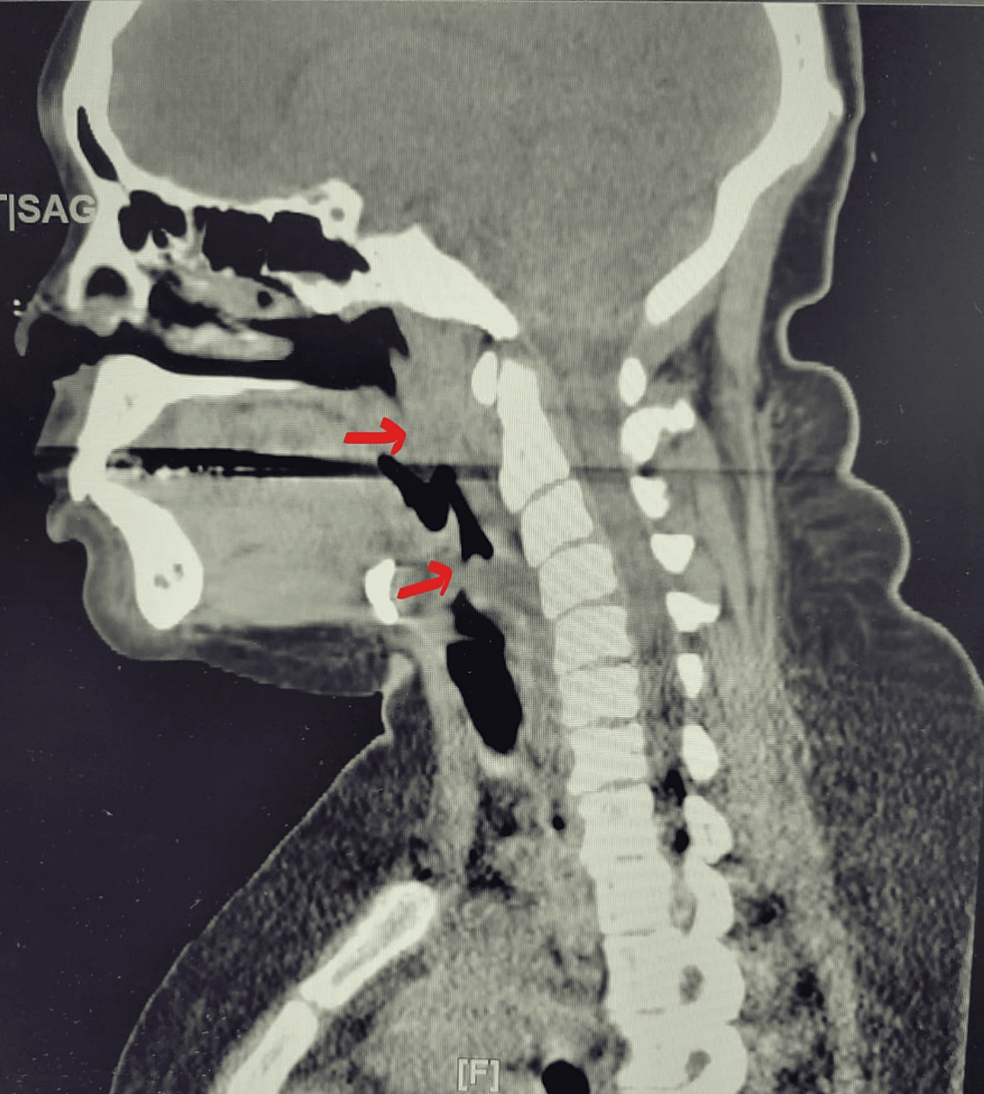
Effacement of the airway originating at the level of the hyoid bone with near-complete effacement at the level of the thyroid cartilage and reconstitution of the airway at the level of the inferior portion of the cricoid cartilage The top arrow shows effacement of the airway at the level of the hyoid bone. The lower arrow shows complete effacement at the level of the thyroid cartilage.

Repeat CT neck showed effacement of the airway originating at the level of the hyoid bone with near-complete effacement at the level of the thyroid cartilage and reconstitution of the airway at the level of the inferior portion of the cricoid cartilage (Figure 2).

Ear nose throat (ENT) surgery was consulted and suggested to proceed with laryngoscopy. A discussion about prophylactic tracheostomy was initiated but the patient refused. The patient refused laryngoscopy and requested to increase diphenhydramine to 75 mg on multiple occasions. She also requested to place a midline and start IV fluids. She threatened to refuse all treatments and leave the hospital against medical advice. On multiple occasions, she endorsed feeling that her tongue is swollen, and throat is closing, even though there was no stridor or wheezing on examination, no tongue or uvula edema noted, and she was able to speak in full sentences.

Psychiatry was consulted due to the history of multiple intubations and no severe pathology on objective exams and laboratory results. Factitious disorder was considered, as the patient has control over her treatment environment, only talking to people she felt like talking to, taking medications of her choice, and frequently changing hospitals and physicians. 

Finally, the patient agreed to proceed with flexible laryngoscopy, which showed a normal pharynx and larynx with normal, functional vocal cords. The patient was discharged in stable condition and was offered to follow up at a psychiatry clinic, however, she refused and preferred to go to another hospital.

## Discussion

Factitious disorder is a psychological condition that can manifest in a panoply of ways in a healthcare setting. Although rare, FD via angioedema is a manifestation of somatoform disorder related to anaphylaxis [1].

After reviewing our patient’s case, there was no significant evidence to diagnose her condition as angioedema caused by unknown or organic circumstances. From the psychological evaluation, there was no evidence of her trying to find any external reward, ruling out malingering. However, her history of 19 endotracheal intubations suggests that she is willing to endure invasive procedures to assume a sick role. This case of a young female presenting with multiple episodes of angioedema with negative laboratory workup, allergy panel, and vocal cord swelling with a concurrent diagnosis of major depressive disorder and generalized anxiety disorder suggests that FD should be considered the most likely differential diagnosis. Her normal serum C1 esterase inhibitor and C4 levels on multiple admissions is a very specific data point supporting FD [1].

Proper diagnosis, management, and resolution of FD is an extraordinary challenge in modern healthcare. This difficulty is compounded by rare forms of FD such as FD presenting with angioedema, as it would be catastrophic for physicians to miss an acute and life-threatening illness. Due process is needed to ensure the best possible outcome for our patient [1]. However, this can be detrimental in cases of FD, as the amount of attention needed for angioedema is necessarily high. Therefore, this may incentivize patients with FD to continue to seek treatment despite it being unnecessary. This is especially problematic in the era of coronavirus disease 2019 (COVID-19), where medical resources are limited due to the large surges of patients presenting to hospitals. Our patient began presenting regularly to our hospital system in March of 2020. Intubations, treatments, and a bed for her meant one less for someone suffering from COVID-19 or other morbid conditions. She underwent a multitude of workups at various times, which was tedious and took many hours of work, redirecting hospital resources. The cost, not only monetarily but also of resources, effort, and brain power, can potentially deter physicians from diagnosing patients with factitious disorder. While we never want to abandon any patient who seeks help, proper diagnosis and resource stewardship are crucial in the era of COVID-19.

Discussion with patients must be done conscientiously since patients can become emotional with any confrontation challenging their perception of their disease [2,4]. If there is a high degree of suspicion of FD, using placebos followed by allergens should be done. Additionally, early psychological evaluation, multidisciplinary integration, and consistent communication can help reduce hospitalizations [5]. Angioedema is a potentially life-threatening condition [6]. However, as FD is a psychological issue deeply rooted in a patient’s perception of self, psychotherapy is not necessarily curative, which presents another difficulty in proper treatment [1].

In the USA, the prevalence of factitious disorder/malingering is 10% to 30% of patients seeking compensation, with an estimated cost of $20 billion per year. Also, unnecessary treatments and investigations may place patients at risk of iatrogenic complications and even death. A better understanding of the disorder and its outrageous financial and emotional costs is encouraged [7].

## Conclusions

This case provides an important example and assists in promoting awareness of patients with possible FD secondary to self-inflicted anaphylactic reactions. Patients who are willing to submit to numerous invasive procedures in order to fill a sick role endanger themselves, utilize a vast number of crucial resources, and put both the patient and healthcare workers at risk of unnecessary treatments that can cause additional and unexpected consequences. Although rare, it is vital to examine patients with multiple, suspicious hospitalizations and raise the idea of factitious disorder. A compassionate and understanding subsequent approach can be beneficial to both the patient and the healthcare system and lead to the best possible outcome.
